# A Study to Explore the Professional Conceptualization and Challenges of Self-Management in Children and Adolescents with Lymphedema

**DOI:** 10.1089/lrb.2018.0076

**Published:** 2019-04-22

**Authors:** Christine Moffatt, Aimee Aubeeluck, Elodie Stasi, Meadbh Macsweeney, Fabienne Mourgues, Hélène Pourquier, Chantal Lapointe, Sandrine Mestre, Isabelle Quéré

**Affiliations:** ^1^ School of Social Sciences, University of Nottingham Trent, Nottingham, United Kingdom.; ^2^ Department of Vascular Medicine, CHU Saint Eloi, University of Montpellier, Montpellier, France.; ^3^ School of Health Science and Medicine, University of Nottingham, Nottingham, United Kingdom.; ^4^ Center of Research of Immunopathology and Rare Diseases—Coordinating Center of the Network for Rare Diseases of Piedmont and Aosta Valley, Turin, Italy.; ^5^ Lymph Clinic, Cork, Ireland.; ^6^ CHU Sainte Justine, Université de Montréal, Montréal, Canada.

**Keywords:** lymphedema, lymphoedema, self-management, self-efficacy, emotional burden, complex decongestive therapy (CDT)

## Abstract

***Background:*** The aim of this study was to explore the professional experience of caring for children and adolescents with lymphedema and to explore the way in which they understand and implement self-management strategies and the influence of their own self-efficacy beliefs on this process.

***Methods and Results:*** Participants were recruited during an educational camp for children with lymphedema. Three individual semistructured focus groups were undertaken in English, French, and Italian with simultaneous translation. Data were analyzed using interpretative phenomenological analysis (IPA). Analysis of the data produced three superordinate themes: professional concepts of self-management, professional practice, and redefining the cornerstone of lymphedema care. An additional seven subthemes were as follows: readiness to self-management, professional perspectives on self-management, defining success and treatment failure, emotional burden, traditional views on complex decongestive therapy, new ways to practice, and sole practitioner versus multidisciplinary teams.

***Conclusions:*** The purpose of the study was to explore the challenges professionals face when introducing self-management to children and adolescents with lymphedema and their parents and to explore their own sense of self-efficacy in approaching this. The research allowed in-depth discussion about the ways they conceptualize self-management and faced professional challenges. The research highlighted the need to define what is considered an acceptable outcome within a complex and uncertain condition and the self-management strategies that are needed to support this.

## Psychosocial Impact on Families

Research indicates that lymphedema impacts on the lives of children and their families, with greater difficulties occurring during adolescence.^1^ However, little is known of the challenges of providing daily treatment and its impact on family life. Even less is known of the professional challenges of managing children and adolescents within the context of different health care systems and service models. The cornerstones of lymphedema management have been traditionally defined as a set of techniques (complex decongestive therapy [CDT]) involving care of the skin, exercise, compression, and manual lymphatic massage, a specialized form of remedial massage.^2^ Treatment has been split into two phases. The first involves an intensive treatment, provided by professionals, that is often delivered for several weeks in which the goal of treatment is rapid removal of fluid and stabilization of the skin. The second phase is defined as maintenance in which in an ideal world, parents take on daily responsibility for delivering a modification of these techniques.

## Self-Management in Lymphedema

A systematic review of activities or treatments that patients used in lymphedema self-management was undertaken using Orem's self-care deficit theory.^3^ The review concluded that self-management does not include professionally delivered treatment and that control of swelling is only one outcome of treatment with symptom control of high importance. Richard and Shea support the importance of managing symptoms and gaining a level of control over the condition.^4^

The dichotomous view that professional treatment of lymphedema is separate from patient self-management is difficult to reconcile as both frequently coexist. Traditional techniques, such as compression therapy, are based on professional procedures and are adapted and taught to children and parents. Procedures are largely based on adult recommendations without clear articulation of what should be adapted despite different challenges. Moffatt and colleagues in a study of CDT in adults highlighted the clash between professional training in CDT and the inability to predict which patients would respond to treatment.^5^

## Aim

The aim of this study was to explore the professional experience of caring for children and adolescents with lymphedema, a rare disease, and to explore the way in which they understand and implement self-management strategies and the influence of their own self-efficacy beliefs on this process. The study was nested within a larger study to explore the enablers and barriers to self-management in children, adolescents, parents, and professionals.

## Research Setting

Professionals were recruited during an international educational camp run under the auspices of the International Lymphoedema Framework (ILF) for children with lymphedema in Turin, Italy (2017). The sample included a mix of clinical teams and individual practitioners from Italy, France, and southern Ireland. All professionals recruited were involved in the care of the children and adolescents attending the camp.

## Ethics

Approval for this study was given by the University of Nottingham Faculty of Medicine and Health Science Ethics Committee. As it was not an intervention study, no formal ethical approval was required in Italy. The research was performed in accordance with international research standards, including the Helsinki Declaration.^6^ All participants gave informed consent and were made fully aware of their right to withdraw from the study if they wished to do so. All study information was translated and back-translated into the different languages to ensure accuracy with English.

## Methods

All professionals from each country attending were invited to attend a focus group and could participate in their native language, or a second language if they preferred. Group size was determined by the numbers wishing to attend and therefore the size was not limited.

Two researchers moderated the groups, which were simultaneously translated so that they could be undertaken in their native language (English, French, and Italian). The researchers wore headsets to ensure they understood the translation. The moderators of the focus groups undertook other research roles during the camp.

### Procedure

Following consent, participants were invited to introduce themselves and explain their roles in relation to the children attending the camp. The Focus groups were semistructured with initial questions focusing on the following: the ways in which they selected children to attend the camp, how they viewed self-management in lymphedema, the professional challenges they faced, and the way in which they addressed these issues. Following this general discussion, questions were responsive and tailored to each individual group. Finally, the researchers summarized the main points raised and asked whether participants' thoughts had been summed up adequately and whether anything had been missed. All focus groups were audiotape recorded and lasted between 1 and 1.5 hours. Immediately following each focus group, both researchers met and completed a reflective diary of the event to capture initial impressions that may have been lost.

### Data analysis

Each focus group session was transcribed verbatim and interpretative phenomenological analysis (IPA) used.^7^ The first transcript was read several times, the left-hand margin being used to annotate what was interesting and significant about what the respondents said. Once this had been carried out for the whole of the first transcript, attention was returned to the beginning of the transcript with the right-hand margin annotated with emerging themes. These themes were then taken back to the original transcript to validate their existence within the text. Emergent themes were then listed on a sheet of paper and studied for connections between them. All the themes were clustered together to produce a set of superordinate concepts. As the clustering of themes emerged, they were continually checked in the transcript to make sure they were evident in the primary source material.

The next stage was to produce a coherently ordered table of themes to establish which most strongly captured the respondents' issues or concerns. The clustered themes were given names that represented their overall superordinate theme and an identifier was added in each instance to aid the organization of the analysis and facilitate checking back to the original transcript. During this process, themes were dropped if they did not fit well into the emerging structure or were not very rich in evidence. Themes from the first transcript were then used to ordinate the analysis of subsequent transcripts. As such, repeating patterns were established, but the emergence of new issues was also recognized. Data from each country's focus group were then compared across the three transcripts to explore the cultural differences emerging.

To determine reliability, an additional researcher undertook independent thematic analysis of the verbatim transcripts. Both researchers discussed the themes and subthemes they had identified, and agreement was sought when meaning was deemed the same but the language used was different so that informed consensus was achieved.

## Results

Participants (*N* = 14) were asked to discuss in as much detail as possible how they viewed self-management for children with lymphedema and their parents and how they addressed this within their practice. Participants' accounts clustered around three superordinate themes: professional concepts of self-management, professional practice, and redefining the cornerstone of lymphedema care, with an additional seven subthemes: readiness to self-manage, professional perspectives on self-management, defining success and treatment failure, emotional burden, traditional views on CDT, new ways to practice, and sole practitioner versus multidisciplinary teams.

Tables 1–3 outline the superordinate themes and subthemes arising from the data from each country with supporting quotes.

**Table 1.** Superordinate Themes (1) and Subthemes (2) with Supporting Quotes (English)

**Professional concepts of self-management (1)**
**Patient readiness to self-manage (2)**
“When you are with the family you need to be able to understand what if going on and if they have any ability to manage things by themselves. This is very important, otherwise, they may not be able to cope with the treatments on top of the load of the condition.” (P1)
“Often the way we ask people to self-manage, we have to be very careful; if we want the patient to self-manage, there has to be also therapeutic support so the interaction is key to self-management.” (T2)
“I have a little questionnaire that I give to make sure that they are ready to take on looking after themselves, to self-manage.” (T1)
“I try to calm down people who are not self-confident with the families immediately. I really wait for a long time just to be sure I am cautions, I don't give any self-efficacy tools immediately until I am confident.” (P1)
**Professional perspectives on self-management**
“I think self-management is the most important thing when you have a little bit of time you must encourage this.” (T1)
“Self-management is to be alert to the skin condition, red flags, infection after that exercise, and what kind of exercise we can do and self-assessment before and after the exercise, how is the lymphoedema react with the exercise I am doing? Is it working? Is it making things worse?” (T3)
“Bandages, they don't like them, parents they find it hard to find the time to do them and they don't like to do them, so I worry that I am going to lose them, so I keep it simple to start with and look at self-management after.” (T1)
“The main thing I think about with self-management is usually how I am going to help the parents with the baby, how will they be able to use the equipment, and how will I be able to help them to self-manage through this period of their life? Usually they come from very far and they have been through difficulties as part of the diagnosis and the parents are very afraid, so a big part of self-management is helping them to deal with their anxieties and try to help them feel that they are in the right place, that they can go home and be safe at home, and if they have any needs they call us.” (P1)
“You have an expectation within self-management that there is a therapist that will work with the self-management.” (T1)
“I think it might be I am thinking that we may have two different schools of thought, one is as soon as you have lymphoedema you must treat it quickly and you have to remove the lymphoedema and from my perspective, self-management has no room there. And the other school come more from the management of a disease you expect to live with and obviously I come from this second school.” (P1)
“I am using my expertise to help them move normally, they are small kids and it is important to help them to walk well. My therapy is all based around getting the kid moving. After that I might think of self-management but at a basic level it is about the kid moving.” (T2)
**Redefining the cornerstone of lymphedema practice (1)**
**Traditional views of CDT (2)**
“My focus used to be for lymphoedema, to decongest, to reduce volume and get them into a stocking and I think I wasn't taught enough.” (T1)
“What I am talking about is training so how much emphasis in school is given to self-management? So much of the training is given to the techniques that actually the time that is given to the context of self-management of the patient is very little.” (T1)
“I think the difficulty in hearing some of the parents this morning is different therapists from different schools and different backgrounds, they will receive different messages.” (T2)
**New ways to practice (2)**
“I think in the beginning we were so focused on the volume decongestion and the treatment and the concept of treatment but now, 15 years later my cornerstones are compression, skin care and exercise.” (T1)
“I think if you change the parameters of what we consider a successful outcome absolutely we will require separate training. So, 160 hours PG training, once you open this box, we will need 160 more hours of whatever, its takes the specialty of working with these patients to a different level.” (T2)
“The concept of compliance vs adherence, that changed my practice so much for me.”(T1)
**Professional practice (1)**
**Defining success and failure of treatment (2)**
“You ask them if they have exercised or moved and they say that intended to join the gym. You ask them if they have worn their garment and there is always a problem, it's too loose, it's too tight, they don't like the creaming.” (T1)
“Sometimes we have patients who are hospitalized from the infection and all we can do is be patient and provide a message about how to take care of themselves.” (P1)
“The reality is people who don't self-manage deteriorate.” (T2)
“I love doing these educational sessions because the patients walk away feeling more knowledgeable and therefore more empowered.” (T1)
“It depends where you are sitting on the table. So if you ask me that in relation to X my version of successful outcome may not match X, X wants to get into a pair of jeans. I know I can get him to another 20% volume reduction but will that get him into jeans? No. Does it keep him safe from cellulitis?. Are we successful? We are not sure.” (T1)
“I accept, it I do, I don't try and change any more, I don't bombard them with the same thing, I just give a little compassion and if there is a problem, if they have a little cellulitis, if they have a larger volume, we'll deal with it, that's all I can do within the limitations.” (P1)
“Sometimes you have to take a step back to just realize that you can be ok with this and maybe it is not perfect but it is good enough.”(P1)
**Emotional burden (2)**
“I have one hour that I know I have this leg to deal with and I understand that it is x that needs most support so but where do I go with this? I am his therapist, I like psychology, I like it I can hear, I can listen, but I am not, I am in a different role, I can't take x and sit for 40 minutes and ask her what can we do to help you? I have 8 more patients to see.” (T1)
“One of my patients doesn't like school, won't do sports for me and that makes me worried.” (T2)
“It is too difficult, maybe we don't, I mean at the end of the day you are just so limited, your focus really is probably on the swelling, because remember we are all coming from different places.” (T1)
“I think the expectations, very unhappy, very unhappy, it is difficult to hear and we have nothing else.” (P1)
“I went to the bathroom and looked in the mirror and I said, he is YOUR patient for the next hour, find a way, find a way to just do this, you feel a failure, look at you, you know how hard you have worked. If patients are not self, sufficient, you see your working practice as a failure so it is a reflection of some sort.” (T1)
“You get used to being everything and it is good to sometimes to burst the bubble.” (T1)
**Isolation versus team (2)**
“Networking has meant when I don't know the knowledge I have people that I can email and talk to and this has changed my self-management advice and language.” (T1)
“Difficulty we have what I see is you have to self-manage yourself as a team and it is impossible so in a way you are treating patients and you are in the position of self-managing alone.” (T2)

*N* = 3 participants.

CDT, complex decongestive therapy; P, physician; T, therapist.

**Table 2.** Superordinate Themes (1) and Subthemes (2) with Supporting Quotes (French)

**Professional concepts of self-management (1)**
**Readiness to self-manage (2)**
“Good self-management is somebody who doesn't need me, doesn't come and see me in hospital, manages to do sport, attends school regularly, and has a social life with friends. That's what it means to me.” (T2)
“Some of the adolescents are very deep and articulate. We talk to the patient about their experiences and how they look after themselves, but it is different for each specific patient.” (T1)
“We meet with the patients during their counselling and depending on their needs we will guide them towards a self-management, self-care situation.” (P1)
“First patients are examined by the doctor to see if they can participate in self-management counselling sessions.” (T1)
“As far as I am concerned, we always pay attention to the family, but the family do not always want us to intervene and want to find ways of managing their own anxieties. In these situations I do not suggest to move forward for self-management.” (P1)
“I have noticed that people have to develop coping mechanisms to enable them to have normal development, the mechanisms that are important are the knowledge to know they are coping and also the relationship they develop with the team, a true partnership that if there is something wrong, they can come, and we will try to help them.” (Psy1)
**Professional attitudes to self-management (2)**
“If I am really honest, self-management has been used because of diminishing resources.” (P1)
“Being able to take care of oneself without any help, the patient understands the disease so they can do things right and acquire their independence.” (T3)
“People who can form a habit and keep on with their treatment. Discharge them. Never, never, never, never.” (T2)
“When I saw them the very first time they were not coping at all, they didn't have any mechanisms, they were lost. They were struggling with no diagnosis, didn't know what was happening, but at the same time they were meeting with people who did know and understood so it was like a miracle.” (Psy1)
“These patients reject any self-management so we, with adult patients we tell them now it is up to you, that is what we tell the adult patients, otherwise it would be useless.” (T2)
“It should really happen in a strict way in a set way and you try and get them to think what would happen if you have some flexibility.” (T3)
“We are here to help the patient, but the patient is the main leading actor and if they don't want they don't want.” (P2)
“What is also very important is that the time for both the child and the mother and they are both very concerned about the disease. They have to see that there are others living with the disease. Not being self-centered—you have to have this experience of sharing with others to change the cycle.” (Psy1)
**Redefining the cornerstone of lymphedema practice (1)**
**Traditional views of CDT (2)**
“We are taught the cornerstones of treatment, these are to be undertaken in a set way and we need to fit this into the treatment time we have for the patient.” (T1)
New ways to practice (2)
“Yes, children with a difference have to learn to grow up with this problem, to put up with it. But if they wish they can become leaders, what I mean is the actor is directed by the film director, the parents and the health care professionals are the directors in this relationship.” (P2)
**Professional practice (1)**
**Defining success and failure of treatment (2)**
“She had to be hospitalized twice because she was not compliant with the treatment she had to go through and we had suggested a social worker and a psychologist, and she refused.” (T2)
“We understand what their volume reduction has been, and we can see if the stockings have been worn out, if they have been used a lot and we also ask direct questions and simply talk with the patient. If there is a problem and if they are not using the stockings, they can be honest and tell us they are not using them.” (P2)
“If the volume is not controlled we try and change the treatment, to adjust it and put into place some self-management models that might suit the patient better. That suits their lifestyle better. Maybe the first treatment we suggested for that patient wasn't the right one for them and if we need to we can also suggest hospitalization to help provide the necessary support.” (P2)
“It is absolutely important to adhere to all types of treatment that is prescribed to the patient, not just medication but also other aspects that are equally important otherwise it doesn't work.” (T3)
“You always go ahead by trial and error and only in that way can you find the best treatment and we should do this for every child.” (P1)
“We are not happy and sometimes it is not always easy to find the right solution for the beginning and I tell people it is a journey that we have embarked upon together and once we find the right key, the right solution for that patient, we are happy, but we really need to be patient.” (P1)
“The thinking is that volume is part of the disease and failure to reduce the volume is always failure of the therapist because we are dedicated to a cure so in a way we are in the same position as the parents psychologically and we are also looking for a cure. We have feelings also, very often we feel sadness and it's hard to deal with this because the professionals do have deep inside of us feelings reminding us of our own limitations.” (Psy1)
**Emotional burden (2)**
“Nonadherence. It is a big huge weight and the main issue.” (T2)
“I feel sad, it makes me sad when I am not able to find a solution because we are really willing to help these children and if it doesn't help, it makes me sad.” (P2)
“It is often the case, you spend a lot of time and it really those who you spend more time, the bond is really close, it is really tight and that makes you even sadder.” (T1)
“I feel sort of frustrated and hopeless in conversations with parents who have difficulties in their financial situation, but we always try and find something we can do.” (P2)
“We have a team organisation that is not formalised. When I see patient, I refer to others when I see a patient is desperate and she says ‘it is not true’ (Laughter) it is like a “psychodrama.” (P2).
“Sometimes it is very heavy for us and we feel the lack of a psychologist, but we try and support where we can when the psychologist is not available.” (T3)
“Help to know in reality what they are dealing with either from the disease point of view or through the dynamics with the professionals.” (Psy1)
“There is no opportunity to breathe and there is huge tension.” (T1)
**Isolation versus team (2)**
“Every professional has their own specific role to play as a specific part of the team and I don't believe in the multidisciplinary team as such. In my experience it is not what I would like but in my experience we can make it work but it is difficult.” (T2)
“We try to care for the patients as a whole and everyone has to take part in this and we try and find the right way to do this. But it important to have a second opinion so we do need to discuss between ourselves. This discussion and joke is typical of our teamwork.” (P2)

*N* = 6 participants.

P, physicians; Psy, psychologists; T, therapist.

**Table 3.** Superordinate Themes (1) and Subthemes (2) with Supporting Quotes (Italian)

**Professional concepts of self-management (1)**
**Readiness to self-manage (2)**
“One thing which is not so scientific is that it depends a lot on the personality. So, our work is to take into account the personality, so we have to stop people who want to do more and push somebody who needs a push. It depends on the personality and life experiences.” (T1)
“I made the mistake of trying to involve the parents too quickly in the treatment. I was too hasty to tell the parents to bandage and self-manage. And the mother got scared and she felt as she was left by herself and abandoned. So today I take more time to understand if the parents really want to engage in this self-management and engage with more patients. I involve them slowly.” (T3)
“Unlike most therapeutic paths where patients get involved at the final steps. I did the opposite—bringing them in at the beginning rather than at the end.” (T1)
“At times I suggest too many new solutions, so I think it is an idea to step back.” (T2)
**Professional attitudes to self-management (2)**
“The children can live in a normal way without any restrictions and following the specific rules. Keeping in mind they have to be extra cautious about certain things.” (T1)
“When we talk about self-management we talk about children being able to do something for themselves. And not to chase the nurses and the staff but to understand what they can do by themselves. They don't always need professional help. They can do something on their own.” (T2)
“We have two kinds of patients. One is relying solely on the health care system and wait for treatment. In my opinion one should be more responsible and not delegate to others what they can do themselves.” (T1)
**Redefining the cornerstone of lymphedema practice (1)**
**Traditional views of CDT (2)**
“We are taught the techniques to use in a certain way.” (T1)
**New ways to practice (2)**
“It is very important to socialize. And share your loneliness. You lose hope like with every rare disease, when you are lonely, you lose hope. Talking to other people about the way they approach the disease you can see the light. When you are in a difficult situation you try to hold on to everything.” (P1)
“What I do when I have a patient under my care I take them for 10 days (children and parents). There is less conflict between them and there is less hostility towards the situation. So they are not afraid to make mistakes. So they have more confidence and they are not shy to talk about things. Then when they come here they are more confident and not so afraid. I tell them how to do the bandages etc.” (T2)
“Physiotherapists spend a long time with the patients compared to a doctor. Hours. When they go to the doctor it is just for 15 min. When they come to us they are liberated, they feel authorized to tell anything and about what the doctor told them and ask all the questions they have.” (T1)
**Professional practice (1)**
Defining success and failure of treatment (2)
“She seemed like a success in the beginning. She had the psychologist. She went back home and told her friends. She was happy. I don't know what happened as she is also someone else's patient. But every time I try to organize something she is ill/has a stomach ache. But I insisted on having her in the camp but it did not help.” (T1)
“Most patients say ‘yes, yes we do the treatment,’ ‘we comply’ and then they return to check with the GP and they have not done it as promised. Only one patient who is not my patient who has a problem with the arm and came here last year and I have seen her again recently, but her arm has gotten worse and I got scared. It is a teenager. It might be linked to her age. She does not do anything.” (P1)
**Emotional burden (2)**
“The mother felt angry, not at me, but at the medical profession because she said ‘if we would have known about these treatments (the child) would not have been in this progressed situation.’ And therefore it was very emotional.” (T1)
“In a certain sense I feel responsible. I am sorry. I would like to understand what is wrong. In reality I got upset, at this point I would be tempted not to call her anymore. I have to think about it. I have to digest it.” (T2)
“We cannot take all the responsibility. We are doing this emotional approach to the problem. We have a holistic approach. It is also a management of the patient. We have difficulties curing chronic patients.” (P1)
“When this affectionate bond is created I also feel responsible. As if they were my children.” (T1)
“We are like a valve. I have not been prepared to deal with all of that. I also have to deal with cancer patients and their emotions. Perhaps we also have problems at home or sick people at home, so sometimes I really struggle with this emotional load.” (T2)
“I try to do my best and I ask a lot to myself. Sometimes I cannot cope with it and I worry if I did the right thing or not. At the end I mean well. Even if I got it wrong and did a mistake I did my best. I feel lonely.” (T1)
“In doing this job I discovered I have difficulty detaching myself. I am always late, and I discovered it is because I cannot detach myself from anybody I feel affection to.” (T1)
“Italian patients listen so much more to what the GP says than what we say. Despite the fact we spend a lot of time with them.” (T3)
“As for children most of the time it is the parents who are talking to us about their story. And at time there are deep things coming out, so difficult stories. That is why we need a psychologist.” (T1)
**Isolation versus team (2)**
“We need a teamwork, a psychologist. We lack the support of psychologists. We are alone. We need to manage all the emotions and feelings. We have to deal with it.” (T1)
“In X we do not have any experienced doctors. Us physiotherapists we are alone.” (T2)

*N* = 5 participants.

P, physician; T, therapist.

## Professional Concepts of Self-Management (1)

### Parent readiness to self-manage (2)

Professionals varied in the methods they used to appraise whether they considered parents and families were ready to engage with self- management. Appraisal methods varied from completion of a questionnaire to interaction over a prolonged time with the family that included assessment by a psychologist. There was overall concern that introducing self-management strategies too quickly could easily overwhelm families who were already facing anxiety and uncertainty over their child's condition.

Readiness was also influenced by the professional's confidence in dealing with high levels of clinical uncertainty in the control of the child's condition and the possibilities of complications occurring due to inappropriate parental decisions. Sole practitioners were concerned about their isolation and vulnerability in making these decisions without access to a multidisciplinary team. Within teams, the decisions concerning whether families were ready to engage with self-management were made by doctors responsible for the child's care and endorsed by psychologists rather than by a therapist. This differs from the experience of sole practitioners who were often therapists and were therefore facing these decisions alone. Some stressed the importance of parents supporting each other in self-management because of their unique experiences of having a child with a similar condition. There was recognition that this included parental solutions to problems that may differ from the advice given by professionals.

### Professional perspectives on self-management (2)

Many professionals defined self-management in relation to the ability of the family to be independent from continuous professional care and for the child to have a normal life. However, there was a deep concern that families had a direct access to professional advice when they needed this and that they remained connected to the services rather than being discharged.

Therapists discussed self-management in relation to the techniques such as exercise and compression that are part of CDT, and the challenges of parents taking on these roles. Adherence to these procedures was often described in a causal relationship to treatment success or failure and was attributed to the level of parental engagement. Attitudes to the strictness that parents should adopt in self-management varied considerably and suggested this was linked to the professional ability to tolerate uncertainty. Some reported that continuous adherence to a set of techniques was directly linked to the control of swelling, while others were more relaxed in their assessment of this.

The integration of self-management into providing professional care was seen to be time-consuming and required continuous engagement with families. There was a strong belief in the need for a therapeutic relationship based on trust that could not always be rapidly or easily established. The challenges of having adequate time for families were discussed by professionals from all disciplines and countries.

Traditional methods of training about the treatment of lymphedema left therapists feeling ill-equipped to address psychosocial issues. This was exacerbated by the challenge of integrating this aspect within clinical visits, which were focused on providing clinical techniques, often leaving little time to address the wider issues. Physicians also discussed the difficulties of making decisions about the role parents could play and the creative solutions they had to take. This included admission of the child to hospital if parents were not coping.

The concepts of self-management were understood differently and ranged from paternalistic attitudes to following professionally prescribed treatment regimens to flexible approaches that sought solutions with the families. Self-management was often conceptualized as a set of techniques or procedures that parents would have to undertake rather than by a broader definition. Professionals from all countries recognized the importance of providing correct information for parents who were facing an array of information that was complex, rapidly evolving, and could be incorrect or misleading, leading to escalating parental anxiety.

Some approached self-management in a formal way and provided training, while others viewed this more fluidly. The camp was viewed as a multifaced intervention that would enable parents to cope better with their child's situation as well as breaking their sense of isolation. The professional views on self-efficacy and the ability of parents to succeed with self-management were complex and integrally linked to the judgments they made on successful or failed outcomes of care.

## Professional Practice (1)

### Defining success and failure of treatment (2)

The findings from this study indicate that stability of swelling measured by limb volume remains the dominant outcome of treatment across all services and countries. However, the different professional responses to interpreting these measurements suggest that they are seeking a “good enough” outcome. This is more nebulous to define and influenced by many clinical and psychosocial issues within the patient and family and within professional belief systems.

Poor control of swelling in children who are clinically stable without frequent complications and who appear to be developing normally is viewed with less concern than those who are not. Experienced clinicians working within teams appear to show greater tolerance to this than sole practitioners. There is evidence that professionals struggle with families in whom there is a clash in the goals of treatment between the child and parent, particularly if these expectations appear unrealistic. The importance of the child or adolescent leading a normal life was of critical importance but appeared difficult to quantify. Attitudes to adherence to treatment indicate that some professionals define poor outcome with reference to poor adherence.

### Emotional burden (2)

Professionals describe a high emotional burden. During all focus groups they became distressed and expressed their surprise at this. The emotional burden was a result of a complex array of issues and was present in those working within teams and by sole practitioners. The level of uncertainty concerning many of the children's future was difficult for many to manage.

Professionals felt overwhelmed by parents in whom there was an expectation of cure or stability and in those who expressed frustration with the limitations of the current knowledge and treatment options. Relationships with children and parents were often deep and led to some being concerned of an overattachment and an inability to manage when treatment outcomes were not as they wished or when parents or children rejected help and sought other solutions.

Professionals as well as children and parents appeared to be seeking a “cure” and were therefore facing their own limitations and feelings of impotence. Teams appear to share the dilemmas together describing this as a “psychodrama,” whereas individual practitioners speak of isolation and being emotionally overwhelmed and ill-equipped to cope. The ability to tolerate ambiguity and uncertainty is a dominant factor underpinning the emotional responses.

## Redefining the Cornerstone of Lymphedema Practice (1)

### Traditional views of CDT (2)

The perspectives held by professionals about the role of CDT in children and adolescents were complex. There was some evidence that this was influenced by the underpinning professional background. Examples of this were seen in physiotherapists who talked of the importance of maintaining mobility. Nurses and physiotherapists were deeply embedded in the methods of CDT training they had received. Physicians also discussed CDT and the need to adapt the protocols they used in “a trial and error basis.” They appeared more able to step back from CDT and consider the wider issues but expressed the burden of overall responsibility for the child. The burden of finding an effective adaption that children and parents could manage was evident in all focus groups irrespective of the country. Teams were prepared to share how these dilemmas impacted on the team and could result in tension that was not usually discussed. Access to a psychologist was discussed as being a vital component to assist both the patient and teams to understand the complex issues they were facing.

### New ways to practice (2)

The experience of the camp offered new ways for professionals to consider delivering care and support for families. The opportunity to connect with families during social events further developed the relationships they held with them in a nonhierarchical way.

Sharing the difficulties as well as learning together appeared to be a cathartic experience for sole practitioners as well as teams, and was not readily available in clinical practice. While parents sought the advice of professionals during the camp, there was also evidence of how professionals valued the opportunity to discuss difficult challenges together, as well as developing new approaches to activities such as aqua aerobic sessions.

Therapists working alone explored the difficulties they faced in influencing physicians who did not listen to their recommendations or prescribed incorrect treatment approaches. They faced the dilemma of parents who had been wrongly advised on sole treatments such as manual lymphatic massage use without compression. They discussed how much value there would be in providing ways to support themselves through networks and clinical supervision.

Professionals were challenged to explore how problems such as mosquito bites and the fear of infection were of much greater clinical significance than they appreciated. Hearing the stories from parents about the control of their child's swelling challenged their assumptions about how they defined “normality” and the significance of the problem to the family.

An underpinning ethos behind the camp is the ability to break the cycle of isolation felt by families and an ability to empower them with the strategies to manage more effectively. The research with professionals indicates a mirror image of the need for support for professionals who are facing complex issues and prolonged uncertainty of outcome.

### Sole practitioner versus multidisciplinary team (2)

The research indicates that professionals face many challenges whether they are sole practitioners or working in large teams. Large multidisciplinary teams were able to share the burden between them but also recognized they faced challenges of working effectively together. Psychology support for patients and professionals emerged as an important issue throughout the camp.

Sole practitioners were frequently therapists who expressed the vulnerability they faced of managing the complex patients in their care when they had no access to specialist advice. Despite this, the challenges of managing these patients were evident for all professionals irrespective of where they worked and the country they were based. Professional boundaries of practice vary between countries and the levels of autonomy these roles require. The research would indicate an urgent need to define what is required in specialist practice and service models and define how children and families can best be supported given the national and international variations in health care that will continue to exist.

## Discussion

This study explored the professional experience of caring for children and adolescents with lymphedema, and the ways in which they understood and implemented self-management strategies. Analysis of the data produced three superordinate themes: professional concepts of self-management, professional practice, and redefining the cornerstone of lymphedema care. From these an additional seven subthemes were identified: professional readiness to self-manage, professional perspectives on self-management, defining success and treatment failure, emotional burden, traditional views on CDT, new ways to practice, and sole practitioner versus multidisciplinary teams.

The research showed the difficulties professionals have in conceptualizing self-management and the clash that exists between the traditional views of CDT and self-management strategies. Research has shown that patients with lymphedema navigate a complex path that makes adherence to treatment difficult. The intrusiveness on daily life may contribute to the low levels of adherence reported in adult studies.^8^ This research found that professionals differed in their views on adherence and whether this contributed to unstable swelling and complications.

Multiple studies in lymphedema show that while the traditional primary focus is control of swelling, this is not the only troubling symptom with altered sensations, psychological distress, changes in body image, fatigue, and functional limitations leading to reduced activity.^9–13^ The benefits of adherence to treatment can be expected to therefore influence beyond control of swelling to other aspects of life and are therefore of critical importance for professionals to understand and integrate into clinical practice.

The study found that there is considerable emotional burden from providing care. Central to this is the uncertainty of outcome and difficulties in being able to define the expected pathway for children and the consequent impact this placed on practitioners. The importance of uncertainty in managing chronic illness has been known for many decades. Uncertainty is the inability to determine the meaning of illness-related events and occurs in situations where the decision maker is unable to assign definite values to objects and events and is unable to accurately predict outcomes due to a lack of information.^14^ Because uncertainty indicates the situation is vague and ill defined, there is potential for many diverse evaluations and conclusions about the uncertainty. While the focus is frequently on the outcomes of care for patients, nevertheless, the professional response to this uncertainty has been shown in adult patients with lymphedema to cause professional anxiety and an inability to define a set of reasonable outcomes in a complex patient population.^5^

A central concept in self-management is self-efficacy, which has been defined as the confidence to carry out behaviors that are necessary to reach a desired goal. Self-efficacy is enhanced when patients succeed in solving patient-identified problems. People's belief that they can take control over their condition is of special relevance to self-management.^15^ Research has shown that motivational, cognitive, affective, and physiological processes affect self-efficacy beliefs and alter health outcomes such as symptoms, physical and emotional well-being, and enhanced social activities.^16^ The perception people have of their self-efficacy also affects their thought patterns and ability to manage stress and depression.^17^ People's belief systems have been shown to affect both acute and chronic illness outcomes, including the degree of benefit they perceive from a health intervention.^17^

Lymphedema management (CDT) is typical of many conditions developed in an era when an acute illness model still predominated, and concepts and structures continue to influence the way in which care is approached.^18,19^ Lymphedema may be defined as a chronic disease, however, treatment with CDT is based more on an acute model of care with interventions delivered during acute exacerbations. This may influence the ineffectiveness of health care systems designed to treat acute disease rather than chronic disease.

Sound management of lymphedema requires participation of patients and families at most levels of health care, from understanding the disease process through to applying self-management strategies, and requires a functioning participation between the patient, family, and the professionals. This study found that professionals struggled to make decisions about when and how to ask families to engage with self-management, with concern for the parental ability to manage additional burdens of care. The study indicated that professionals from all disciplines struggled with their own self-efficacy beliefs in the face of children and adolescents in whom the future was uncertain. This resulted in a tension between preventing deterioration through ensuring self-management techniques were used to prevent deterioration and an awareness of the burden placed on families.

The educational camp was seen to play a complex role in addressing the issues of engaging parents in self-management and highlighted the diversity of challenges faced by all professionals. Central to future research is the description of self-management practices that can be evaluated for their efficacy and impact on the wide group of stakeholders involved in successful support of children, adolescents, and parents who face this important but neglected condition.

### Study limitations

This study has several limitations. It only represents the views of the professionals who agreed to take part in the focus groups during an international educational camp. As such, they may not reflect the views of others who do not access such camps. It is well acknowledged that professional education in lymphedema practice is highly varied with a lack of evidence in many aspects of treatment. The views of the participants will be influenced by many factors, including their professional background and the way in which they have been trained to asses and treat patients with lymphedema. There are many different service models operating internationally with a variation in roles and responsibilities. Caution should be given to the generalizability of the research, which must be extended to a wider participation. Despite this caveat, the study shows that many of the issues raised were common across the groups, indicating that self-management in this is an important professional issue.

## Conclusion

The purpose of investigating the experience of professionals working with children and families with lymphedema was to give a greater understanding of the challenges they face and their own sense of self-efficacy. The focus groups provided a forum that allowed in-depth discussion about the ways they conceptualize self-management and faced professional challenges. The research highlighted the need to define what is considered an acceptable outcome within a complex and uncertain condition and the self-management strategies that are needed to support this.

## Implications for Clinical Practice

The research has shown an urgent need to address the following issues:

- Develop multidisciplinary services for children in all countries.
- Provide clarity on self-management strategies in lymphedema.
- Define a range of outcome parameters that extend beyond control of swelling to psychosocial health.
- Educate health professionals about the reality of managing a child or adolescent with lymphedema.
- Develop and evaluate low-intrusion self-management programs that incorporate self-efficacy assessment.
